# Decipher the Molecular Response of Plant Single Cell Types to Environmental Stresses

**DOI:** 10.1155/2016/4182071

**Published:** 2016-03-20

**Authors:** Mehrnoush Nourbakhsh-Rey, Marc Libault

**Affiliations:** Department of Microbiology and Plant Biology, University of Oklahoma, 770 Van Vleet Oval, Norman, OK 73019, USA

## Abstract

The analysis of the molecular response of entire plants or organs to environmental stresses suffers from the cellular complexity of the samples used. Specifically, this cellular complexity masks cell-specific responses to environmental stresses and logically leads to the dilution of the molecular changes occurring in each cell type composing the tissue/organ/plant in response to the stress. Therefore, to generate a more accurate picture of these responses, scientists are focusing on plant single cell type approaches. Several cell types are now considered as models such as the pollen, the trichomes, the cotton fiber, various root cell types including the root hair cell, and the guard cell of stomata. Among them, several have been used to characterize plant response to abiotic and biotic stresses. In this review, we are describing the various -omic studies performed on these different plant single cell type models to better understand plant cell response to biotic and abiotic stresses.

## 1. Use of Plant Single Cell Types to Study Plant Response to Environmental Stresses

**Figure 1:**
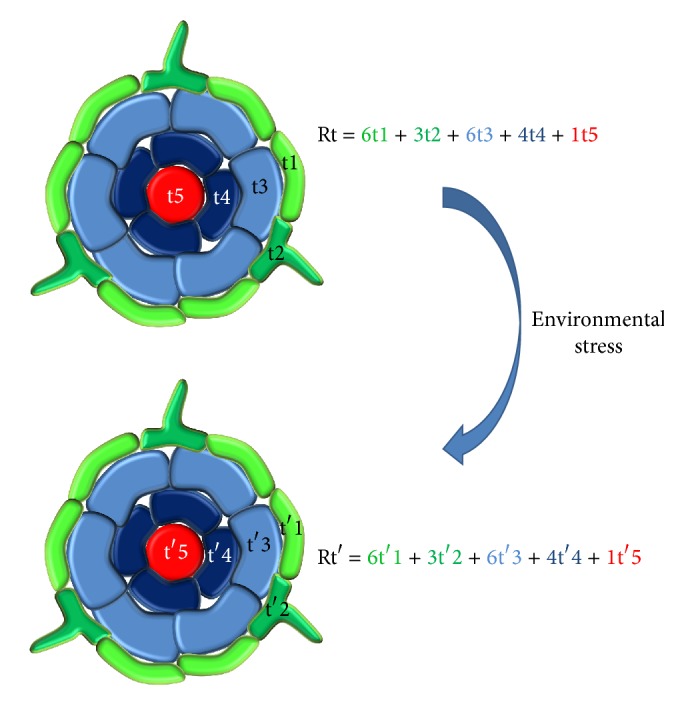
Root transcriptomic response to a stress (Rt and Rt′) is the sum of the individual responses of each cell type composing the root (t1 to t5; t′1 to t′5). Because plant cell transcriptomes are different between cell types, the cellular complexity of plant roots is not suitable to characterize gene networks. A single cell type model must be used to better characterize plant gene networks.

The multicellular complexity of the samples collected to characterize plant response to environmental stress is a major limitation to clearly depict the contribution of each cell type composing the sample in response to the stress. In other words, -omic studies at the level of plant organs reflect the average response of the different cell types composing the organ (Figure 1). In order to fully understand the exact contribution of each plant cell type in regulating plant response to environmental stresses, the transcriptome, epigenome, proteome, metabolome, and interactomes (e.g., protein-protein and protein-DNA interactions) of each plant cell type composing the sample and their changes in response to environmental stresses should be independently characterized [1]. For instance, the characterization of the transcriptional response of the soybean root hair cell to rhizobial inoculation allowed the identification of almost two thousand differentially expressed genes [2]. This single plant cell type analysis represents a significant improvement compared to previous studies describing few hundred genes differentially expressed in root sections in response to rhizobial inoculation [3, 4]. Ultimately, the integration of these various datasets will lead to a global understanding of the molecular adaptation of plants to environment changes through the precise characterization of transcriptional regulatory networks [5]. Currently, the construction of these networks in response to environmental stresses is highly dependent on the nature of the samples used to collect biological information. For instance, working at the level of entire organs cannot depict the specific networks existing in each cell type composing the organ supporting the idea to study plant biological networks at the level of single cell types. Accordingly, this review highlights the recent progress in the field of plant adaptation in response to both biotic and abiotic stresses at the level of single cell types (Table 1).

## 2. Isolation of Plant Single Cell Types

The isolation of plant single cell types is limited by the cell wall, which provides both rigidity and structure to the plant and acts as a first barrier against pathogenic organisms. To overcome this difficulty, different strategies were applied to isolate various cell types. These technologies include the use of cell sorting laser capture microdissection [6–9], sorting of single plant cell types upon cell type-specific GFP labeling and protoplastization [10, 11], and the application of the INTACT (Isolation of Nuclei TAgged in specific Cell Types) method which includes the labeling of single plant cell nuclei with a biotinylated nuclear envelope protein before their isolation using streptavidin-coated magnetic beads [12, 13]. Other methods have been applied to access in large quantities easily accessible single plant cell types such as cotton fiber and root hair cells [14–16]. More recently, an innovative methodology named Meselect which combined both a mechanical and an enzymatic treatment of the plant cells has been applied to isolate leaf epidermal, vascular, and mesophyll cells [17]. Lu et al. (2015) also developed another methodology allowing the isolation of generative cells (GCs), the sperm cells (SCs), and the vegetative nuclei (VN) from tomato pollens [18]. In this method GCs, SCs, and VN were isolated from germinated tomato pollen grains and growing pollen tubes and purified by Percoll density gradient centrifugation. Microscopic examination of fluorescein diacetate-stained samples confirmed the purity of GCs and SCs, respectively. Propidium iodide staining was used to confirm VN integrity.

Currently, only a limited number of single plant cell types have been isolated in quantities compatible with the application of -omic technologies. The most noticeable examples are the cotton fiber, pollen cells, and root hair cells [14, 16, 19–22]. Various root cell types from the model plant* Arabidopsis thaliana* have also been isolated preliminary to their molecular analyses including response to environmental stresses [23–26]. Our understanding of the biology of the plant female gametophyte which is composed of antipodal, central, egg, and synergid cells is also beneficiating from single cell type analyses [27].

## 3. Single Cell-Specific Transcriptomes in Response to Biotic and Abiotic Stress

Compared to other -omic datasets, plant single cell type transcriptomes and their changes in response to environmental stresses are currently the most complete. For instance, multiple studies have characterized the transcriptomic profile of* Arabidopsis thaliana* root cells and their response to abiotic stresses including nutrient deprivation (i.e., iron and sulfur), salinity, and low pH values as well as in response to stress-signaling plant hormones such as abscisic acid [24, 25].

Among root cells and across plant species, the root hair cell is likely the best transcriptionally characterized single cell type based on the ease to isolate them from the rest of the root. The root hair transcriptome has been characterized across different plant species including* A*.* thaliana* [13, 26, 28–31],* Glycine max* (i.e., 451 root hair specific transcripts characterized [2, 32]), and* Medicago truncatula* (i.e., 49 root hair specific transcripts characterized [33]). In legumes, this single plant cell type was also recently used as a model to study plant cell response to biotic stress because it is the first cell type infected by rhizobia, the nitrogen-fixing symbiotic bacteria [2, 33]. 219 and 79 soybean and* Medicago* genes were, repetitively, transcriptionally regulated in root hair cells in response to rhizobia including many genes functionally characterized for their role during nodulation [34]. Another plant single cell type model recently used to characterize the transcriptional changes occurring in response to pathogenic microorganisms was the* A*.* thaliana* mesophyll cell infected by the oomycete* Hyaloperonospora arabidopsidis* [35].

In addition to respond to various biotic stresses, plants are also constantly interacting, responding, and adapting to various abiotic stresses. Our understanding of those interactions is also benefiting from a single plant cell type transcriptomic approach. For instance, Sarah Assmann's group performed a transcriptomic analysis of* A*.* thaliana* guard cells in response to abscisic acid, a plant hormone acting on plant water conservation. 909 genes were specifically regulated in response to ABA in guard cells [36]. Plant resistance to heavy metal has also been investigated at the level of single plant cell types. For instance, trichomes are known to sequester heavy metals such as cadmium. Accordingly, a comparative transcriptomic analysis was conducted in* Nicotiana tabacum* trichomes in response to cadmium treatment [37]. Together, taking advantage of the specific biological function of single plant cell types, their transcriptomic analysis has the potential to reveal new plant regulatory genes in response to biotic and abiotic stresses due to the gain of sensitivity of the analysis. For instance, the barley *β*-extension* EXPB7* gene which was initially identified based on its differential expression in root hair cells in response to drought stress has been demonstrated to confer a better drought adaptation to the plant [38].

## 4. Characterization of the Proteomic Response of Single Plant Cell Type to Environmental Stresses

Proteins are the active molecules in the cells. The quantification of their relative abundance is critical to understand plant adaptation to environmental stresses. However, single cell type proteomes are challenging to establish because of the limited quantities of plant material available [39–41]. In addition, their posttranslational modifications are also affecting protein function and should logically be characterized at the level of single plant cell types.

A first effort in the establishment of single cell type proteome was the characterization of the pool of proteins in mature pollens. Pollen cell proteomics have been studied in different plant models such as* Arabidopsis thaliana* and* Oryza sativa* [42–45]. In* Arabidopsis thaliana* 130 differentially expressed proteins involved in pollen germination and pollen tube growth were identified via proteomic analyses [46]. Ultimately, proteomic analyses led to the establishment of the first protein reference map of mature pollen in* Arabidopsis* using two-dimensional gel electrophoresis (2DE), matrix-assisted laser desorption/ionization time of flight (MALDI-TOF), and electrospray quadrupole time of flight-mass spectrometry (EQ-TOF-MS) [47]. In maize, a comparative proteomic analysis allowed the characterization of differentially expressed proteins involved in pollen tube development and plant defense [48]. Among those proteins, several participate in pollen resistance to environmental stresses. For instance, proteomic analysis in* Arabidopsis* pollen helped to reveal the role of the ABI1 phosphatase 2C as a negative regulator of ABA signaling [49]. Similarly, in response to osmotic stress, glucose regulated (GRPs) and LEA-like* A*.* thaliana* proteins were strongly induced to protect the cells [47, 50, 51].

Trichome has also been subject to various proteomic analyses across different plant species such as* Artemisia annua*,* Arabidopsis*, and tobacco [52–54]. The establishment of the trichome proteome in* Arabidopsis thaliana* confirms the important role of this single cell type in sulfur metabolism and detoxification to enhance plant defense mechanisms [55]. Upon the identification in 1543 proteins in tobacco leaf trichomes [56], several enzymes also related to the detoxification including glutathione-S-transferase (GST), ascorbate-glutathione cycle enzymes, superoxide dismutases (SOD), cytosolic Cu/Zn SOD, and peroxidases were characterized in response to oxidative stress [56, 57]. The functional categorization of the* Arabidopsis* trichome proteome based on gene ontology (GO) terms also confirmed the role of trichomes in plant adaptation to abiotic (cold, temperature, drought, and heavy metal) and biotic stresses [54]. Similarly, in tobacco, proteins were also identified for their role in biotic stress responses such as chitinases and glucanases [56].

The guard cell proteome revealed the abundance of proteins involved in signaling, membrane transport, glycolysis, photosynthesis light reaction, and fatty acid biosynthesis [58]. Using isobaric tag for relative and absolute quantitation (iTRAQ) technology, several ABA-response proteins were identified in* Brassica napus* and* Arabidopsis* guard cells [59, 60]. In* B*.* napus*, 66 ABA-dependent and 38 ABA-decreased proteins were reported to have a special function in calcium oscillation, ROS reaction, photosynthesis, and signaling [59].

Comparative proteomic analyses in soybean also led to the identification of several root hair specific proteins differentially accumulated in response to* Bradyrhizobium japonicum* inoculation including more than 100 heat shock proteins involved in protein folding and stress responses [61, 62]. To provide a more complete view about the changes in the proteome of soybean root hair cells in response to rhizobium, Nguyen et al. (2012) also established its phosphoproteome and identified 273 root hair specific phosphopeptides regulated in response to* B*.* japonicum* infection [63].

## 5. Metabolomic Response of Single Plant Cell Types to Environmental Stresses

Similar to the other -omic approaches, the analysis of the cell type-specific metabolomes is affected by multicellular complexity of the tissues selected. In addition to their diversity, metabolomic analyses also suffer from the low concentration of many metabolites supporting the need for single plant cell type metabolomic approaches.

Microcapillary method is applied for sampling plant single cells content by using the oil-filled glass microcapillaries mounted on the micromanipulator [64–67]. This method benefits from cellular turgor pressure to study plant organ water relations at the single cell level, which allows investigating cellular macromolecules [68, 69]. The combination of the microcapillary method with other physical or chemical analytical methods (e.g., gas chromatography-time of flight-mass spectrometry (GC-TOF-MS) [70], laser capture microdissection (LCM), laser microdissection optionally coupled to laser pressure catapulting (LMPC) [71], and RT-PCR [67, 69]) helps researchers to characterize the metabolome of fully differentiated plant cell types [72]. Combined with the recent improvement of analytical technologies, the quantitative and qualitative analysis of plant single cell type metabolomes would provide new insights into the environmental stress responses of plant cells.

Various technologies are commonly used for metabolomic profiling including infrared spectroscopy [73], nuclear magnetic resonance (NMR) [74–76], mass spectrometry (MS) and gas chromatography-MS (GC-MS) [77, 78], matrix-assisted laser desorption/ionization (MALDI) [79], capillary electrophoresis coupled with laser induced fluorescence detection (CE/LIF) [80] or mass spectrometry (CE/MS) [81, 82], CE-negative electrospray ionization-MS, and electrospray ionization (ESI) [83]. These techniques vary in speed, selectivity, and sensitivity.

Several metabolomic studies at the level of single plant cell types have been described. For instance, focusing on the epidermal bladder cell (EBC), a specialized trichome cell from* Mesembryanthemum crystallinum* known to be morphologically altered under salt stress [84], Barkla and Vera-Estrella (2015) characterized their metabolomic response to salinity [85]. Comparing* M*.* crystallinum* EBC metabolomic salt-response with the metabolomes of other salt-tolerant plant species [86, 87], specific classes of metabolites enhancing plant adaptation to high salinity such as sugars and sugar alcohols have been identified. Similarly, having the goal to enhance plant resistance in response to salinity and heavy metals, metabolomic analyses revealed the accumulation of sulfur and glutathione in* Arabidopsis* and tobacco trichomes [88].

In the* Arabidopsis thaliana* and* Vicia faba* guard cells, several metabolomic studies revealed the relationships existing between the accumulation of flavonoids, reactive oxygen species, abscisic acid, nitric oxide, and auxin as important components of the signaling cascade controlling the stomatal movements in response to osmotic stresses and pathogenic organisms such as* Pseudomonas syringae* [89–94]. For instance, the increase in phenolic and flavonoid compounds in the* A*.* thaliana* guard cells provides an additional protection against pathogens, insects, and UV-B radiation [90]. Lipidomic analysis of* Commelina communis* and* A*.* thaliana* guard cells also revealed the role of fatty acids in the stomatal response of plants to both abiotic and biotic stresses [93, 95].

Plant single cell type metabolomic response to biotic stresses has also been established such as the soybean root hair metabolome and its regulation in response to* B*.* japonicum* inoculation. A total of 2610 root hair metabolites were identified using two biochemical methodologies: gas chromatography-mass spectrometry (GC-MS) and ultraperformance liquid chromatography-quadrupole time of flight-mass spectrometry (UPLC-QTOF-MS) [96]. Among them, 166 were highly regulated in response to* B*.* japonicum* inoculation including various flavonoids, amino acids, fatty acids, carboxylic acids compounds, and trehalose. The latter has been well described for its essential role during the nodulation process and, more specifically, its role on survival of the soybean symbiotic bacteria [97, 98].

New approaches are currently developed allowing the noninvasive analysis of single plant cell metabolome associated with a robust quantification and detection of plant single cell metabolites. Infrared-laser ablation electrospray ionization (LAESI) and UV-laser desorption/ionization (LDI) are two methods minimizing sample preparation and manipulation. The latter does not require an external matrix providing a larger spatial resolution of the single cell [99]. Complementary to LDI technology, LAESI highlights the colocalization of metabolites and metabolomic networks in plant samples such as* Spathiphyllum lynise* and* Aphelandra squarrosa* [100]. The LAESI was also successfully applied to analyze the metabolome of single epithelial cells in* Allium cepa*, the* Citrus aurantium* oil glands, and* Narcissus pseudonarcissus* bulbs [101, 102]. As a conclusion, LAESI and LDI are new noninvasive analytical methods compatible with the analysis of single plant cell metabolome. They represent attractive solutions to image known and unknown metabolomic networks in response to environmental stresses at the level of single plant cells.

## 6. Conclusion

Recent technological advances are now enabling the characterization of plant molecular responses to both biotic and abiotic stresses at the level of single plant cell types. -Omic studies on entire plant organs mask the cell-specific characteristics and lead to a dilution of the molecular changes. Accordingly, the scientific community highlighted the need for single plant cell type approaches to provide a more precise molecular characterization of plants response to the abiotic and biotic stresses. The combination of different molecular approaches and their integration will reveal at a systems level the complexity of plant cell adaptation to the stresses.

For instance, using tomato pollen cell as a model, Lopez-Casado et al. (2012) generated a proteomic analysis using RNA-seq database [103]. Single cell system biology by combination of one or two -omic analyses can also provide a more dynamic model of the interactions between the plant and its environment [104]. Therefore, integrating single cell type-specific proteomes, transcriptomes, and metabolomes would provide a better understanding of plant model regulatory networks in response to environmental stresses [22].

## Figures and Tables

**Table 1:** Various -omic analyses were conducted on different plant single cell types in response to both biotic and abiotic stresses.

Cell type	Omics		
	Transcriptome	Proteome	Metabolome
Trichome	*Nicotiana tabacum *L. cv. Xanthi (pathogenic stress; [37])	*Arabidopsis thaliana* (cold, hormone stimulus, and drought; [55]) * Artemisia annua *L. (dehydration stress, detoxification; [53]) * Nicotiana tabacum* (oxidative stress; [56, 57])	

Guard cell	*Arabidopsis thaliana* (dehydration stress; [36])	*Brassica napus* (ABA response; [59]) * Arabidopsis thaliana* (ABA response; [60])	*Vicia faba L.* (darkness and drought; [89]) * Arabidopsis thaliana* (nitric oxide response and ABA response, pathogenal infection, and UV radiation; [90–94])

Mesophyll cell	*Arabidopsis thaliana *(pathogenal infection; [35])		

Root hair	*Hordeum vulgare *L. ssp.* spontaneum *(drought stress; [38]) * Arabidopsis thaliana *(heat, cold, salt stress, oxidative stress, and abscisic acid stimulus; [28–31]) * Glycine max *(rhizobial infection; [2]) * Medicago truncatula* (rhizobial infection; [33])	*Glycine max* (rhizobial infection; [61–63])	*Glycine max* (rhizobial infection; [96–98])

Pollen, pollen tube	*Arabidopsis thaliana* (heat and osmotic stress; [19, 28])	*Arabidopsis thaliana* (pathogenic infection, oxidative stress; [42, 44–47]) * Zea maize *(oxidative stress; [48]) * Oryza sativa* (pathogen infection, oxidative stress; [43])	

Epidermal cell	*Mesembryanthemum crystallinum* (salinity stress; [88])		*Mesembryanthemum crystallinum* (salinity stress; [85])

Cotton fiber	*Gossypium arboreum *L. (drought stress; [14, 15])		
